# Advancements in the Quest to Map, Monitor, and Manipulate Neural Circuitry

**DOI:** 10.3389/fncir.2022.886302

**Published:** 2022-05-26

**Authors:** Jessica L. Swanson, Pey-Shyuan Chin, Juan M. Romero, Snigdha Srivastava, Joshua Ortiz-Guzman, Patrick J. Hunt, Benjamin R. Arenkiel

**Affiliations:** ^1^Department of Molecular and Human Genetics, Baylor College of Medicine, Houston, TX, United States; ^2^Jan and Dan Duncan Neurological Research Institute, Texas Children’s Hospital, Houston, TX, United States; ^3^Department of Neuroscience, Baylor College of Medicine, Houston, TX, United States; ^4^Medical Scientist Training Program, Baylor College of Medicine, Houston, TX, United States

**Keywords:** neural circuit tracing, cell type-specific, viral genetic technology, calcium/voltage indicator imaging, neurotransmitter/biosensors, chemogenetics, optogenetics, targeted ablation

## Abstract

Neural circuits and the cells that comprise them represent the functional units of the brain. Circuits relay and process sensory information, maintain homeostasis, drive behaviors, and facilitate cognitive functions such as learning and memory. Creating a functionally-precise map of the mammalian brain requires anatomically tracing neural circuits, monitoring their activity patterns, and manipulating their activity to infer function. Advancements in cell-type-specific genetic tools allow interrogation of neural circuits with increased precision. This review provides a broad overview of recombination-based and activity-driven genetic targeting approaches, contemporary viral tracing strategies, electrophysiological recording methods, newly developed calcium, and voltage indicators, and neurotransmitter/neuropeptide biosensors currently being used to investigate circuit architecture and function. Finally, it discusses methods for acute or chronic manipulation of neural activity, including genetically-targeted cellular ablation, optogenetics, chemogenetics, and over-expression of ion channels. With this ever-evolving genetic toolbox, scientists are continuing to probe neural circuits with increasing resolution, elucidating the structure and function of the incredibly complex mammalian brain.

## Introduction

Neural circuits are the functional building blocks of the brain, comprised of the unique inputs and outputs of connected sets of neurons with particular functions. Nodes within circuits are the keystone checkpoints of neural circuits. In other words, nodes represent anatomically and functionally distinct ensembles of cells that integrate and process neural information from many different inputs to dictate the output of that circuit. These complex networks of connectivity are responsible for everything from processing sensory information (Lohse et al., 2020), attaching valence to stimuli that drive motivated behaviors (Reynolds and Berridge, 2002; Root et al., 2014; Al-Hasani et al., 2015), maintaining physiological homeostasis (Atasoy et al., 2012; Madden and Morrison, 2019), learning, and consolidating memories (Ruder et al., 2021; Sharpe et al., 2021).

Initially, studies of the brain relied on gross anatomy, defining large structures at relatively low resolution, without knowledge of cell type (Scoville and Milner, 1957). Coarsely targeted lesions using electrical current or chemicals allowed researchers to impart functional significance to these anatomically distinct brain regions (Foster et al., 2003; Lavond and Steinmetz, 2003). Early studies also used dyes and microscopy to identify and trace neurons, characterize their morphology, and identify connectivity patterns within discrete brain regions (Golgi, 1886). Electrophysiology was used to classify different neurons by their electrical signature, and to record their inputs and outputs (Piccolino, 1997). Additionally, early electron microscopy experiments provided subcellular resolution of synaptic structures, affording an unparalleled but somewhat myopic view of the brain (Gray, 1959). Although groundbreaking in their time, these applications were limited due to their lack of cell-type specificity, and the constraints of static analysis due to the need to harvest tissue.

One of the continued challenges in neural systems research is dissecting circuit function despite incredible interconnectivity. Many neural circuits have multiple, and often redundant, functions. Adding to this complexity, each node within a circuit has numerous inputs and outputs, as well as feedback and feedforward patterns of interconnectivity. For example, in early lesion studies, it was found that the hypothalamus appeared to affect appetite and body weight (Cushing, 1932; Brobeck, 1946; Brooks et al., 1946; Stellar, 1953; King, 2006). As this circuit was dissected further, it was revealed that two molecularly defined cell types (AgRP/NPY and POMC neurons) appeared to work in opposition to each other to drive either appetite or satiation, respectively (Luquet et al., 2005; Aponte et al., 2011; Zhan et al., 2013). Further studies uncovered that these cells express different receptors with different input and output targets and that POMC neurons are even inhibited locally by AgRP neurons (Sohn, 2014; Sternson and Atasoy, 2014; Wang et al., 2015). Currently, it is now appreciated that this circuit influences diverse behaviors outside the scope of feeding, including locomotion and foraging (Huang et al., 2013; Dietrich et al., 2015). Additionally, numerous non-hypothalamic nodes are sufficient and necessary for normal appetite and body weight control, including regions such as the basal forebrain, amygdala, and dorsal raphe nucleus (Campos et al., 2016; Herman et al., 2016; Kim J. et al., 2017; Patel et al., 2019; Bond et al., 2020; Bruschetta et al., 2020; Ye et al., 2022). This example highlights the complexity of the functional circuitry underlying behaviors such as feeding. As the questions regarding neural circuit structure and function become progressively sophisticated, the tools used to answer them must also evolve.

**FIGURE 1:**
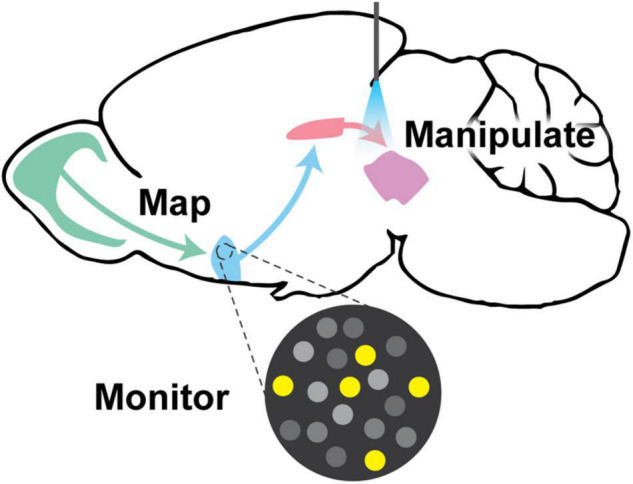
Visual abstract. This review discusses the paramount goals in neural circuitry research, which are to (1) map neural circuit connectivity using viral tracing approaches, (2) monitor circuit function using methods such as electrophysiology and genetically encoded indicator/voltage/sensor imaging, and (3) manipulate neural circuits to interrogate function via targeted ablation, expression of exogenous ion channels, chemogenetics, or optogenetics. All of these approaches require creative genetic tools to implement them in genetically-defined populations of neurons.

Fortunately, the toolbox for interrogating neural circuits has expanded rapidly. More precise intersectional genetic approaches have allowed interrogation of circuits with higher resolution and greater cell-type specificity. Additionally, it is now feasible to label groups of neurons based on activity patterns rather than just molecular markers, which may reveal more information about functional ensembles within neural circuits. This review will highlight cell-type-specific tools for targeting neural circuits, which enable more precise (1) mapping and tracing, (2) dynamic monitoring of neural circuit activity, and (3) targeted manipulation of neural circuits via loss- or gain-of-function strategies to infer function (Figure 1). Foremost, intersectional genetic tools and activity-dependent recombinases are used to target molecularly- or activity-defined neuronal populations. These genetic approaches are fundamental for performing any mapping, monitoring, or manipulation experiment with cell type-specificity. Secondly, with advancements in viral tracing strategies, it is now commonplace to map monosynaptic inputs and outputs of circuit nodes with cell type-specificity. This review will describe the contemporary approaches being used for such labeling strategies. Thirdly, it is critical to monitor neural circuit activity to better correlate function. Advancements in activity recording methods, such as genetically encoded calcium and voltage indicators and neurotransmitter sensors, provide new avenues toward understanding how neural circuits function in awake, behaving animals. Finally, causally interrogating neural circuit function via targeted activation or inhibition of select neurons within that circuit lends critical insight into function. Toward this, increasingly sophisticated tools allow investigators to activate and inhibit neural circuits with approaches that include genetically-targeted cell ablation, chemogenetics, and optogenetics. Neuroscientists still have much to learn about the brain, but with these advancements in the ability to map, monitor, and manipulate neural activity, the field of systems neuroscience is in the midst of an intellectual renaissance.

## Genetic Approaches Toward Achieving Cell-Type Specificity: Site-Specific Recombination, Intersectional Genetics, and Activity-Based Targeting

Historically, brain circuits were investigated using tools that lacked cell type-specificity, such as lesions, pharmacology, and dyes. While this provided critical information about connectivity and function, we now know that each node in the brain is comprised of heterogeneous cell types, whose roles may be functionally distinct. Defining the neuronal constituents that contribute to circuit output is paramount to understanding brain architecture and function (McCulloch and Pitts, 1943). Thus, developing methods to label neurons in a molecularly- or activity-selective manner is imperative.

### Site-Specific Recombination and Intersectional Genetics

**FIGURE 2:**
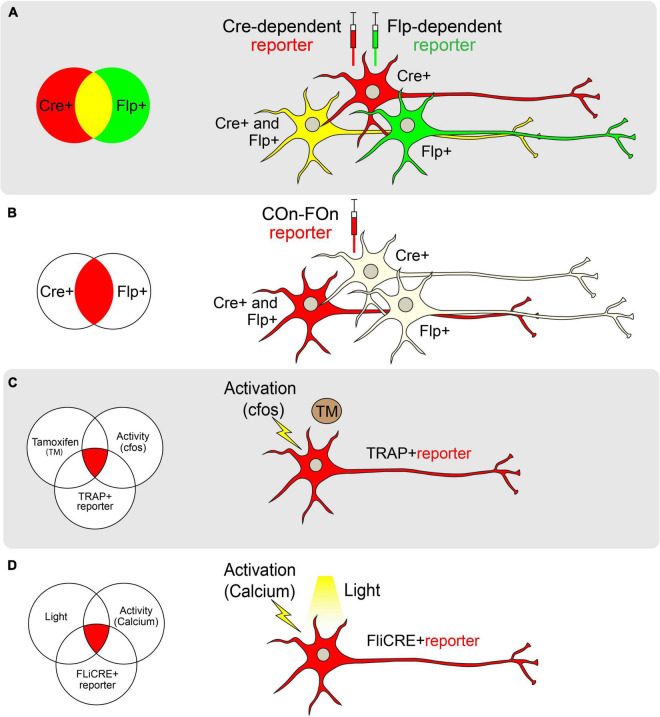
Genetic approaches toward achieving cell-type specificity. **(A)** Schematic of labeling neurons with different reporters using Cre (red) and Flp (green) recombinases separately. Cre and Flp-positive cells are visualized in yellow. **(B)** Schematic of labeling neurons with both Cre and Flp recombinases using the Con-Fon reporter, which is designed to be expressed only when both Cre and Flp recombinases exist. **(C)** Schematic of TRAP. The reporter of interest will be expressed only when Tamoxifen (TM) and neural activity (as indicated by *cfos* expression) exist in a TRAP + neuron. **(D)** Schematic of FLiCRE. When light and neuronal activity (as measured by an increase in calcium concentration) exist simultaneously, neurons will express the FLiCRE reporter of interest.

Neuronal subtypes have been best defined and manipulated by their unique gene expression profiles (Colosimo et al., 2004; Gray et al., 2004; Baumgardt et al., 2007; Flames and Hobert, 2009; Winden et al., 2009). This has partially been made possible through the discovery and creative implementation of site-specific recombination (SSR). SSR affords the ability to selectively target neurons via controlled expression of Cre- and Flp-recombinases (Figure 2A; Dymecki, 1996; Dymecki and Tomasiewicz, 1998; Chai et al., 2000; Awatramani et al., 2003; Branda and Dymecki, 2004; Gong et al., 2007; Anastassiadis et al., 2009; Meinke et al., 2016). Such recombinases can be utilized to excise, invert, or conditionally express genes of interest via the “Lox-Stop-Lox” (LSL), “FRT-Stop-FRT” (Kuhlman and Huang, 2008), or “double-inverted orientation” [DIO; FLEx or FLEx (FRT)] methods (Atasoy et al., 2008; Schwarz et al., 2015). Of these, the DIO approach has proved extremely useful when paired with viral vectors to conditionally express genetic reporters or actuators in a region- or cell-type-specific manner. Additional recombinase/sequence pairs have recently been engineered to expand such approaches, including Dre/rox, VCre/VloxP, and SCre/SloxP (Anastassiadis et al., 2009; Suzuki and Nakayama, 2011). Similar to the Cre/lox system, these pairs can be used to delete, invert, and conditionally express genes of interest depending on the relative orientation of the rox, VloxP, or SloxP sites.

In cases where a neuronal population of interest lacks a single unifying molecular marker, multiple pairs of recombinases and sequences can be combined in an intersectional manner (Figure 2B). The Lox and FRT sites in these systems are oriented such that a gene will only be expressed in cells that express both Flp and Cre (for example, Frt-Stop-Frt followed by a FLEXed gene of interest; Dymecki et al., 2002; Awatramani et al., 2003). To reduce the possibility of leaky reporter expression due to stop codon read-through, an alternative intersectional strategy involves placing Lox and FRT sites at intronic sequences that divide a gene of interest, allowing for tighter control of gene expression. Moreover, intronically engineered sites and their adjacent exons can be oriented to facilitate genetic alterations only in cells that express both recombinases (ex. Cre-On, Flp-On or “COn-FOn”; Figure 2B), only one recombinase but not the other (ex. Cre-On/Flp-Off or Cre-Off/Flp-On), or in all cells except those that express both recombinases (ex. Cre-Off/Flp-Off; Fenno et al., 2014, 2020). Additionally, three-factor-dependent constructs have been generated (ex. Cre-On/Flp-On/VCre-On) to achieve even tighter genetic targeting control (Fenno et al., 2020). Such intersectional strategies also allow for the combination of spatially-restricted and cell type-specific drivers for genetic targeting. For example, using a retrogradely-transported Flp (Tervo et al., 2016) in combination with a cell type-specific Cre driver endows the ability to target cells that express a specific marker and project to a defined anatomical location [as in Rossi et al. (2021)].

While many experiments require the use of transgenic approaches to trace circuits or express actuators, engineering transgenic mice can be time-consuming, costly, and have limited experimental applications. The advent of viral genetics to express conditional genetic constructs (via the DIO or similar method) allows one to perform virtually any experiment in a cell type of interest if a Cre-driver is available (Haggerty et al., 2020; Nectow and Nestler, 2020). Viruses also provide better spatial control when targeting specific brain regions. Adeno-associated viruses (AAVs) are the most commonly used viruses in neuroscience. The simplicity of genomic structure, ease of packaging, and relatively non-pathogenic nature have made engineered AAVs ideal for genetic tracking, activity monitoring, and neuromodulation (Saleeba et al., 2019). Moreover, AAVs have selective tropism endowed by their capsid protein, which can be tailored for tissue of interest. For example, capsids such as AAV DJ/8, 2, and 9 are readily used for brain-specific expression (Haggerty et al., 2020; Nectow and Nestler, 2020). Due to the vast experimental possibilities brought about by viral genetics, teasing apart circuit function has been limited largely by the availability of recombinase-based genetic drivers that specify given cell populations. However, the advent of single-cell RNA sequencing is rapidly expanding to reveal potential gene candidates whose expression may serve as reliable and precise markers of different neuronal populations (Zeisel et al., 2015; Gokce et al., 2016; Chen et al., 2017b; Saunders et al., 2018; Tepe et al., 2018; Rossi et al., 2019; Hashikawa et al., 2020; Wallace et al., 2020). This novel information will provide a much larger repertoire of recombinase-expressing driver mice, a critical tool in the age of viral genetics.

### Activity-Dependent Methods for Accessing Neuronal Ensembles

In some instances, single genes – or even sets of genes – are unable to adequately characterize the function of neuronal populations, which may operate as molecularly heterogeneous ensembles. Therefore, it is sometimes desirable to characterize neurons in an activity-dependent rather than a genetically-dependent manner. To this end, elegant methods have recently been developed to target neuronal populations based on their activity (DeNardo and Luo, 2017). These methods co-opt immediate-early genes such as *Fos* (Greenberg and Ziff, 1984) and *Arc* (Lyford et al., 1995) to drive recombinase expression in active neuronal populations.

Targeted Recombination in Active Populations (TRAP; Guenthner et al., 2013; DeNardo and Luo, 2017) is one such technology in which a tamoxifen-inducible Cre-ER(T2), has been inserted downstream of a *Fos* promoter. In the presence of tamoxifen, active cellular populations expressing *Fos*, and therefore Cre-ER(T2), permit translocation of Cre recombinase to the nucleus to facilitate the expression of a Cre-dependent reporter (Guenthner et al., 2013). Similar models exist that use *Arc* to drive Cre-ER(T2) expression (Guenthner et al., 2013; Figure 2C). A variation of this technology, termed the Tet-tag system, uses a tetracycline transactivator domain downstream of the *Fos* promoter (Reijmers et al., 2007). In this approach, tetracycline-controlled transactivator protein (tTA) binds to the tetracycline responsive promoter element (TRE) to drive gene expression. In the presence of tetracycline, tTA cannot bind the TRE, and target gene expression is dampened. To use this system, tetracycline must be continually supplied in chow. Upon cessation of tetracycline provision, active neurons produce tTA, allowing the transcription of downstream reporters in a temporally-precise and activity-dependent manner (Reijmers et al., 2007).

One of the major limitations of both the TRAP and Tet-tag systems is the potential for the temporal lag in accurately capturing real-time neuronal activation. Since TRAP relies on the systemic administration of tamoxifen (or similar analogs), active neurons may be labeled throughout a 24-h time window. Similarly, due to the slow time course of tTA activation following the removal of doxycycline, the Tet-Off system also captures cells over extended times (Reijmers et al., 2007; Guenthner et al., 2013). Thus, more temporally precise labeling methods have been sought-after. One such method called “CANE” (capturing neuronal ensembles) utilizes a transgenic mouse expressing destabilized avian-specific receptor TVA under the control of the *Fos* promoter (Fos-2A-dsTVA; Sakurai et al., 2016). The dsTVA is fused with a degradation signal so that TVA closely mirrors Fos expression patterns. Upon delivery of pseudotyped lentivirus or rabies virus, neurons actively expressing Fos may be tagged for tracing or manipulation experiments (Sakurai et al., 2016). Other methods that tag activated cells based on calcium signals rather than immediate early gene expression and drug delivery have been shown to be even more temporally constrained. For example, CaMPARI, a photoconvertible genetically encoded calcium indicator, labels neurons only when both calcium levels are high and light is delivered to cells, identifying activated neurons on the millisecond timescale with spatial precision (Fosque et al., 2015). Similarly, FLiCRE (Fast Light and Calcium-Regulated Expression) is a method that labels activated cells at the transcriptional level (Kim et al., 2020; Figure 2D). It not only tags activated cells following a calcium influx but also enables the direct manipulation of the tagged cells via the concomitant light-induced expression of Cre. Collectively, these novel activity-driven targeting approaches allow researchers to screen for neural circuits underlying behaviors of interest. This is useful when a molecular marker for a region/behavior is not known, or when more than one molecular marker may contribute to a particular behavior.

## Mapping Neural Circuits: Viral and Multiplex Neural Tracing Strategies to Dissect Circuit Anatomy

One of the primary objectives in neural circuit research is to create maps that label neurons within a node, as well as their inputs and outputs. These maps are a critical first step toward understanding circuit function, providing information on cellular morphology, and ultimately, informing the creation of a brain-wide connectome. Initially, circuits were traced in a non-cell type-specific manner with small organic compounds or dyes. These conventional tracers – including lectins (PHA-L; Gerfen and Sawchenko, 1984) and dextran-amines (DAs; Gimlich and Braun, 1985) allowed researchers to label the neuronal membrane, visualizing the soma along with its axonal projections. Other compounds were discovered to label a neuron in a retrograde fashion, being taken up by axon terminals and transported to the soma of a neuron. Such compounds included Horseradish peroxidase (HRP; Kristensson and Olsson, 1971; Kristensson, 1977), Cholera toxin subunit B (CTB; Stoeckel et al., 1977), hydroxystilbamidine (FluoroGold™; Köbbert et al., 2000), and retrobeads (Katz et al., 1984; Katz and Larovici, 1990). Although conventional tracers robustly label neurons and provide glimpses into their complex morphology, they are not sufficient to reveal patterns of connectivity between molecularly defined cell types. As a result, viruses – which enable cell type-specificity and transsynaptic labeling – have largely replaced many conventional tracing methods.

### Viral Tracers

Viruses provide many advantages over conventional tracers. Foremost, viral tracers may be engineered as conditional vectors compatible with diverse genetic markers, permitting cell type-specificity. Second, diverse promoters with varying strengths can be used to modulate expression levels or drive cell type-specificity. Lastly, viruses can be engineered to cross single synapses (Kuypers and Ugolini, 1990; Xu et al., 2020). Delivery of viral vectors using stereotaxic injections affords both temporal and spatial specificity, while also obviating the need to generate transgenic mouse models for genetic labeling experiments, so long as the recombinase driver of choice already exists. The following will give an overview of viruses used for both retrograde and anterograde labeling of neural circuits (Table 1).

**TABLE 1:** Properties of commonly used conventional (white) and viral neural tracers (blue).


	Name	Genome type	Onset (transport)	Duration	Transsynaptic labeling	Toxicity
	
**Anterograde**	HSV-129	dsDNA	Hours	Mice die 3–5 days after injection	Yes (Monosynaptic: H129-ΔTK-tdT)	H129-ΔTK-tdT causes toxicity in 3–5 days
	AAV1, AAV9	ssDNA	2–3 weeks	Months	Yes (AAV1 is transsynaptic in GABAergic/ glutamatergic neurons)	Minimal
	CTB		By 24 h	1–2 months	No	Minimal
	PHA-L		1–3 weeks	Several weeks	No	Minimal
	DAs (10 kda)		6–14 days	Several weeks	No	Minimal
	WGA/WGA-HRP		3–4 days	–	Yes	Minimal
**Retrograde**	HSV	dsDNA	Hours	Usually 5–7 days	Yes	Dependent on strain and replication competence
	Pseudorabies virus	dsDNA	2–3 weeks	Months	Yes	Dependent on strain and replication competence
	CAV-2	dsDNA	3–7 days	Months	No	Limited
	Rabies virus	ssRNA	About 2 days	Months	Yes (Monosynaptic: RABV)	Dependent on strain and replication competence
	AAV2-retro	ssDNA	1–2 weeks	Months	No	Minimal
	CTB		By 24 h	1–2 months	No	Minimal
	FluoroGold		7–10 days	Months	No	Minimal
	DAs (3 kDa)		10–15 days	Several weeks	No	Minimal
	WGA/WGA-HRP		3–4 days (WGA-HRP is more sensitive)	–	Yes	Minimal
	HRP		5–7 days (Slightly longer than WGA-HRP)	–	No	Minimal

*Lanciego and Wouterlood (2011), Nectow and Nestler (2020), Vercelli et al. (2000), and Xu et al. (2020).*

#### Retrograde Viral Tracers

**FIGURE 3:**
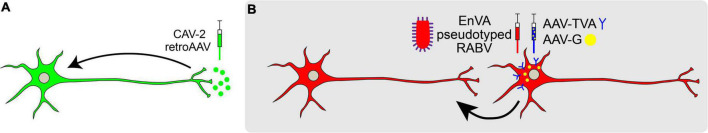
Retrograde viral tracing strategies. **(A)** Non-transsynaptic retrograde tracing using retro-AAV or CAV-2. **(B)** Transsynaptic retrograde tracing using pseudotyped rabies virus is used to trace monosynaptic connections. Rabies G (RB) is provided *in trans* and neurons are pseudotyped with TVA receptor to endow cell-type specificity. Once a neuron expressing TVA and RG is infected with RABV expressing EnvA, the RABV may jump across one synapse in a retrograde manner.

Retrograde viral tracers label inputs into designated populations of neurons (Figure 3A). HSV-1 was one of the first neurotropic viruses used to transfer genes into specific brain regions via stereotaxic injection (Carlezon et al., 1997; Lilley et al., 2001). While favoring retrograde transport, its ability to travel both anterograde and across the synapse rendered this virus limited in its utility (Ugolini, 2010). Thus, viruses that exclusively travel either retrogradely or anterogradely were sought. This led to the adoption of Pseudorabies (PRV; a misnomer since the virus actually belongs to the herpes virus family) and Rabies virus (RV; Martin and Dolivo, 1983; Ugolini, 1995; Enquist, 2002).

Since their discovery, PRV and RV have been widely used to infect the CNS of rodents and non-human primates for retrograde, transsynaptic labeling (Kuypers and Ugolini, 1990; Xu et al., 2020). However, results from these initial experiments proved challenging to interpret, as monosynaptic versus polysynaptic inputs were not discernable. Additionally, viral toxicity caused cell death. To address this problem, pseudotyped RV (RABV) was engineered to label the monosynaptic inputs of genetically and anatomically defined populations (Wickersham et al., 2007). This engineered RABV has a deletion of the endogenous envelope glycoprotein (RG), which is required for viral assembly and transsynaptic transport. By selectively supplying RG *in trans* to source-cells, RABV can replicate and cross one synapse, but no further. Furthermore, pseudotyping RABV with the avian leucosis virus coat protein EnvA restricts RABV to infect neurons that express the cognate TVA receptor, endowing greater control with cell type-specificity (Etessami et al., 2000; Arenkiel, 2011; Figure 3B). Using this approach, it is possible to trace different cell types within the same brain region, quantifying and comparing mapped inputs between targeted source cell types (Do et al., 2016).

Other types of viruses are also capable of revealing monosynaptic inputs to a node of interest. These retrograde viral tracers include canine adenovirus and modified capsids of AAV. Unlike the rabies virus, these viruses are not transsynaptic. Canine adenovirus type 2 (CAV-2) has high neuronal specificity and selectively infects axon terminals of neurons instead of the soma and dendrites, which makes it a powerful tool for mapping inputs to various brain regions (Soudais et al., 2001; Lavoie and Liu, 2020). For example, the use of CAV-2-Cre in conjunction with Cre-dependent fluorescent reporters allows for efficient mapping to targeted neuronal populations (as in Herman et al., 2016). Also, recent advances in AAV capsid engineering endow directionality-specific transduction. Of these, the rAAV2-retro capsid drives retrograde transport of AAV similar to CAV-2, being taken up by synaptic terminals and transported to the soma of the transduced neuron (Tervo et al., 2016). However, one caveat to both retro-AAVs and CAVs is that they may exhibit selective tropisms. For example, some studies have shown that CAV-2 more efficiently transduces certain neuronal populations compared to retro-AAV (Kakava-Georgiadou et al., 2019), while others have shown that retro-AAV is more efficient than CAV-2 for other neuronal populations (Tervo et al., 2016). Thus, potential biases in tropism may somewhat limit the utility of these viruses in certain brain regions or for targeting different cell types.

#### Anterograde Viral Tracers

**FIGURE 4:**
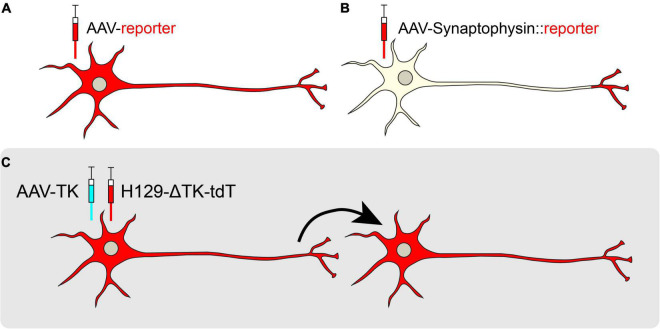
Anterograde viral tracing strategies. **(A)** Non-transsynaptic anterograde tracing using expression of a cytosolic or membrane-bound fluorescent reporter. **(B)** Non-transsynaptic anterograde tracing using expression of Synaptophysin fused to a fluorescent reporter. **(C)** Transsynaptic, monosynaptic tracing using the H129-ΔTK-tdT system. When TK is provided *in trans*, the H129 reporter virus may jump across one synapse in an anterograde manner.

In addition to mapping the inputs, it is also critical to map circuit node outputs, informing the next step in the wiring diagram. Many fluorescent markers labeling either the cytosol or neuronal membrane will label axon terminals, however, it may be difficult to differentiate axon terminals from fibers of passage using these markers (Figure 4A; Xu et al., 2020). A better way to perform anterograde viral tracing is to tag synaptic vesicle proteins with fluorescent markers. For example, synaptophysin::GFP selectively labels presynaptic axon terminals (Li et al., 2010). By conditionally expressing these fusion proteins, one may trace projections in a cell-type-specific manner (Figure 4B). However, close appositions visualized from synaptophysin tracing do not necessarily demonstrate functional synapses (Saleeba et al., 2019). Thus, functional connectivity studies must be performed to validate these projections, as described later in the review. Alternatively, GFP Reconstitution Across Synaptic Partners (GRASP) is a method to visualize anatomically-defined synaptic connections (Feinberg et al., 2008). GRASP relies on the targeted expression of complementary GFP fragments tethered to plasma membrane carrier proteins to detect the proximity of two cells. When two cells form functional synapses, split-GFP fragments reconstitute a functional, fluorescent GFP molecule at the point of contact, labeling the synapse (Feinberg et al., 2008; Kim et al., 2011). For example, when AAV-expressing pre-GRASP was targeted to the auditory cortex and post-GRASP to the inferior colliculus, it was revealed that these two regions are synaptically connected, as indicated by GFP puncta at the point of contact (Song et al., 2018).

Several other tools exist for transsynaptic anterograde mapping. Although most of the HSV-1 families are known to spread selectively in the retrograde direction, the H129 strain moves in the anterograde direction, crossing synapses to downstream target cells (Dix et al., 1983; Zemanick et al., 1991; Wojaczynski et al., 2015). Recently, a modified version of H129, H129-ΔTK-tdT, was developed for monosynaptic anterograde tracing. This genetically modified H129 virus has a deletion in the thymidine kinase (TK) gene that is replaced by a tdTomato reporter. Without TK, the H129 virus cannot replicate in neurons, but supplementation of TK from a helper virus allows H129-ΔTK-tdT to spread polysynaptically, labeling post-synaptic target neurons (Lo and Anderson, 2011; Zeng et al., 2017; Figure 4C). In a recent study, AAV-TK-GFP was stereotaxically targeted to the posteromedial thalamic nucleus (VPM) of wild-type mice, and H129-ΔTK-tdT was delivered 21 days later. Post-synaptic tdTomato was observed in cortical layer IV of the visual cortex (V1), revealing a novel connection from the VPM to V1 (Zeng et al., 2017). A polysynaptic version of H129 (H129-G4) has binary tandemly-connected GFP cassettes inserted into the H129 genome, allowing this variant to move anterogradely in a polysynaptic manner with robust labeling capable of revealing intricate neuronal morphologies (Zeng et al., 2017). However, since H129-G4 demonstrates polysynaptic spread, it may be difficult to discern the direct outputs of a particular node. Finally, capsid engineering has also produced AAVs capable of transsynaptic anterograde tracing. AAV1 is a novel capsid that endows preferential transport to postsynaptic neurons (Zingg et al., 2020). However, while AAV1 transsynaptically spreads in both glutamatergic and GABAergic neurons, it does not spread to neuromodulatory cell types, limiting its utility (Zingg et al., 2020).

### Multiplexed Mapping Methods

**FIGURE 5:**
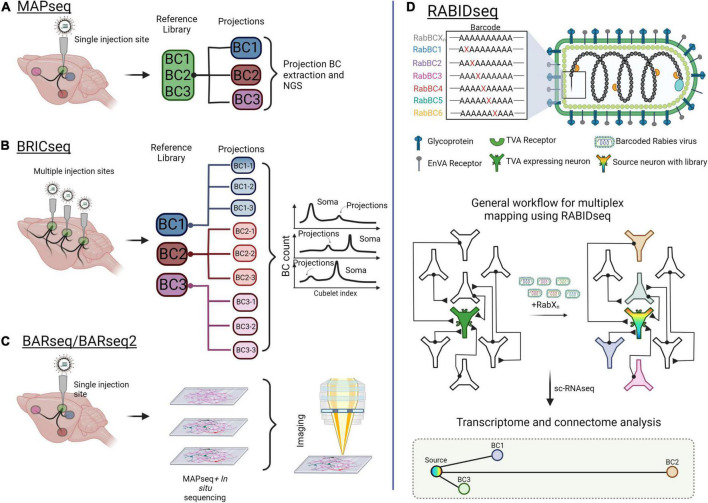
Mapping brain-wide connections with multiplexed, barcoded viral tools. **(A)** Multiplexed Analysis of Projections by Sequencing (MAPseq) uses barcoded viruses to identify projections from a single injection site. Barcodes are extracted from the injection site and from axons at the projections. **(B)** Brain-wide individual animal connectome (BRICseq) uses the same barcoded viral library strategy as MAPseq but for multiple sites in the brain. Different “zipcodes” are designated to a spatial location within the brain and small dissections of tissue are removed for analysis. Soma and projections are then determined by the total makeup of barcodes in the projections compared to soma. **(C)** Barcoded anatomy resolved by sequencing (BARseq) is a technique that combines MAPseq and *in situ* sequencing to maintain the physical position of neurons and their axons within the brain. **(D)** Rabies barcode interaction detection followed by sequencing (RABIDseq) uses rabies virus and sequencing to gather transcriptome data on mono-transsynaptic partners. Created with Biorender.com.

Mapping neural circuits using fluorescent reporters combined with confocal microscopy remains a powerful method for navigating the brain. However, to identify connectivity with single-cell resolution, multiplexed mapping and sequencing approaches have evolved (Figure 5). For example, MAPseq (multiplexed analysis of projections by sequencing) and BRICseq (brain-wide individual animal connectome sequencing) are capable of surveying thousands of neurons in a single experiment (Kebschull et al., 2016; Han et al., 2018; Huang et al., 2020). These approaches utilize the Recombinant Sindbis virus, which rapidly achieves very high expression levels, to express unique mRNA barcodes in individual source cells. These barcodes spread throughout each source cell, including its axonal projections. Both the source area and downstream projections are then micro-dissected, dissociated, and sequenced. After sequencing, barcodes from projection areas are matched to the source area to reveal singe-cell projection patterns (Sun et al., 2021; Figures 5A,B). Recently, Gergues et al. (2020) used MAPseq to map projections from the ventral hippocampus (vHPC). They labeled vHPC neurons with a library of random RNA barcodes, examined 7 different target brain regions, and differentiated projection-specific populations of vHPC neurons (Gergues et al., 2020). One caveat with MAPseq/BRICseq is that the precise positions of individual cell bodies are lost due to tissue dissociation. To overcome this limitation, BARseq (barcoded anatomy resolved by sequencing), combines MAPseq with *in situ* sequencing to maintain cellular spatial localization (Chen et al., 2018, 2019; Figure 5C). BARseq has been applied to map projections from the mouse auditory cortex to 11 areas of the whole brain, confirming the laminar organization of the three top classes of projection neurons in the auditory cortex (Chen et al., 2019).

Other multiplexed mapping methods identify inputs through retrograde transport of barcodes. One such approach is Connect-seq, which determines the molecular identities of individual upstream neurons in a defined circuit by combining retrograde viral tracing and single-cell transcriptomics (Hanchate et al., 2020). Implementing this strategy, Hanchate et al., successfully targeted Cre-dependent PRV infection to corticotropin-releasing hormone neurons within the paraventricular nucleus of the hypothalamus (PVN). Flow cytometry was then used to isolate the upstream inputs, and single-cell RNA-sequencing (scRNA-seq) was used to determine the identities of these inputs (Hanchate et al., 2020). In a similar way, RABID-Seq utilizes pseudotyped rabies virus to label and deliver barcodes to presynaptic inputs in a cell type-specific manner, which may then be identified through scRNA-seq (Clark et al., 2021). Toward this, RABID-Seq has been used to identify axon guidance molecules as mediators of microglia-astrocyte interactions that promote CNS pathologies (Clark et al., 2021; Figure 5D).

In summary, these multiplexed input and output mapping approaches provide unprecedented resolution in the creation of accurate wiring diagrams, providing transcriptomic analyses that are lacking in other contemporary viral tracing approaches.

### Clearing Methods for Whole-Brain Neural Circuit Mapping

As neural tracing strategies have evolved, so too have the microscopes and imaging technologies necessary to visualize them. A major obstacle to visualizing deep brain tissue is signal distortion due to light scattering and spherical aberrations. Consequently, imaging long-range projections were effectively impossible. To mitigate these challenges, emerging optical techniques such as CUBIC (Susaki et al., 2014; Murakami et al., 2018; Matsumoto et al., 2019), CLARITY (Chung et al., 2013; Tomer et al., 2014), ScaleS (Hama et al., 2011, 2015), and SeeDB (see deep brain; Ke et al., 2016) “clear” brain tissue. By removing lipids in whole-brain tissue while leaving proteins and nucleic acids intact, tissue effectively becomes transparent, allowing one to image three-dimensional brain architecture or brain-wide neural projections with minimal light scattering. When used in combination with light-sheet microscopy or similar imaging platforms, intricate circuit networks may be visualized in three dimensions, across the entire brain (Newmaster et al., 2021).

## Monitoring Neural Circuits: Measuring Circuit Dynamics Via Electrophysiology and Genetically-Encoded Imaging Techniques

Once a circuit has been mapped, observing and quantifying circuit activity is necessary to better understand its function. Monitoring circuit activity using both traditional *ex vivo* and more recent *in vivo* methodologies provides information on circuit connectivity and action potential dynamics and correlates circuit function with a particular behavior, sensory perception, or cognitive task. One of the first methods for monitoring circuit activity was recording the electrical activity in the nerve tissue of a dissected frog leg Galvani (Piccolino, 1998). This discovery led to the birth of electrophysiology – the study of the intrinsic electrical properties of cells (Rubaiy, 2017). Electrophysiological techniques record activity dynamics with single-cell resolution, but they are relatively low-throughput in nature. Additionally, while neuron firing patterns can be used to classify certain cell types *post hoc*, these methods overall lack cell type-specificity. Recent advances have increased the throughput and cell type-specificity of electrical recordings. Furthermore, optical approaches, including genetically-encoded calcium/voltage indicators and neurotransmitter/neuropeptide sensors, have gained incredible momentum.

### Electrophysiology

Classical electrophysiology centers around the “patch-clamp” technique, which creates a tight seal on a cell’s membrane using a glass electrode or micropipette to record membrane voltage or current (Neher and Sakmann, 1976). Patch clamping has remained the gold standard of electrophysiology for decades, allowing scientists to measure the properties of ion flow across the membrane and investigate the types of ion channels that regulate the electrical activity of cells. However, recent advancements in patch-clamp configuration allow increased flexibility and greater throughput recordings including cell-attached, inside-out, outside-out, perforated patch, and notably, the whole-cell configuration (Hill and Stephens, 2021).

In an increasingly multimodal research world, electrophysiology has adapted to interface with other sophisticated techniques, allowing users to measure multiple dimensions of neural activity. For example, patch-clamp electrophysiology has been combined with RNA sequencing in a technique known as Patch-Seq. Patch-Seq permits the collection of transcriptomic data after performing whole-cell electrophysiology recordings, as patched cells are drawn into a glass capillary and used for single-cell RNA sequencing (Bardy et al., 2016; Cadwell et al., 2016; Chen et al., 2016; Földy et al., 2016; Fuzik et al., 2016; Lipovsek et al., 2021). Using Patch-Seq, both neuronal activity and gene expression patterns are revealed with single-cell resolution. Indeed, this technique has been used to describe the diversity of neuronal subtypes within the mouse motor cortex, as well as to provide insights into the diversity of neuronal populations in the dorsal striatum (Muñoz-Manchado et al., 2018; Gouwens et al., 2020; Scala et al., 2021).

While recording the electrical properties of single cells has been critical for understanding the basic properties of individual neurons, interrogating the activity of interconnected circuits requires recording from multiple cells simultaneously. Toward this, extracellular recordings using wire or silicon-based electrodes measure action potentials in multiple parallel neurons. By using multiple geometrically arranged electrodes, or multielectrode arrays (MEAs), it is now possible to record many neurons simultaneously from one probe and analyze their summed electrical activity, or local field potentials (LFPs; Buzsáki, 2004; Einevoll et al., 2013). The past decade has seen the rise of increasingly powerful MEAs such as Neuropixels, a silicon-based probe with up to thousands of independent channels for recording hundreds of neurons simultaneously (Jun et al., 2017). With the advent of these new high-throughput recording methods, it is now possible to interrogate the electrical and synaptic properties of circuits with unprecedented spatiotemporal resolution (Csicsvari et al., 2003; Shobe et al., 2015; Jun et al., 2017).

### Genetically-Encoded Calcium Indicators

Electrophysiology is optimized to measure the activity of neurons with high temporal precision but is limited in its ability to spatially localize the cells being recorded, or to identify their molecular cell type. With the advent of real-time neural activity imaging, activity-dependent fluorescent reporters have been developed to measure circuit dynamics with high spatial resolution in living brain tissue. Genetically Encoded Calcium Indicators (GECIs) are the most widely used activity-dependent fluorescent reporters. GECIs are proteins that bind cytosolic calcium and exhibit increased fluorescence with calcium influx, a proxy for neuronal firing, thereby translating neural activity into spatially resolved optical signals (Bootman and Berridge, 1995; Miyawaki et al., 1997; Oh et al., 2019). Since fluorescent activity reporters are genetically encoded, their expression can be targeted to specific populations through conditional viral or transgenic methods (Pérez Koldenkova and Nagai, 2013). While the temporal dynamics are less precise than electrophysiological techniques, advancements in imaging resolution and GECI sensitivity enable monitoring of diverse firing dynamics at high spatiotemporal resolution. Importantly, such imaging approaches facilitate the investigation of firing and anatomical properties of neuronal circuits from diverse brain structures, from the cortex to deep brain nuclei (Hoogland et al., 2009).

GCaMP reporters are currently one of the most widely used families of GECIs (Table 2). Structurally, GCaMP is a fusion protein between GFP, calmodulin (a calcium-binding protein), and the light chain myosin protein, which was rationally designed to facilitate increased GFP fluorescence with the influx and binding of free calcium (Shimomura et al., 1962; Porumb et al., 1994; Tallini et al., 2006; Akerboom et al., 2009). Additional improvements to GECI technology have been made via large-scale random mutagenesis (GECOs; Table 3), or semi-rational mutation strategies (GCaMP5 family) to identify indicators with stronger fluorescence intensity, varied activity kinetics (GCaMP6s-f), and greater dynamic ranges (Zhao et al., 2011; Akerboom et al., 2012; Helassa et al., 2015). Notably, GCaMP6 and GCaMP8 are widely used for their fast kinetics, stability, and high signal intensity. GCaMP8 has a higher signal intensity than GCaMP6, making it well suited for experiments to monitor population activity signals via imaging of the soma, while GCaMP6 is preferred for visualizing activity at dendritic spines (Ohkura et al., 2012a).

**TABLE 2:** Commonly used GCaMP reporters.

Class	Indicator	Emission wavelength (nm)	Description	References
GFP	GCaMP1.3/1.6	510	Slow kinetics for calcium binding; unable to resolve single APs.	Nakai et al., 2001; Ohkura et al., 2005; Reiff et al., 2005; Hendel et al., 2008
	GCaMP2	511	Slow kinetics for calcium binding; unable to resolve single APs.	Tallini et al., 2006; Hendel et al., 2008
	GCaMP3	513	Adequate fluorescence at basal cell [Ca^2+^]. Unable to resolve single action potentials (APs) *in vivo*.	Tian et al., 2009; Akerboom et al., 2013; Redolfi et al., 2021
	GCaMP5A/D/G/K/L	510	Improved dynamic range (increased threefold from GCaMP3). Variants can be chosen according to their properties to fit particular experimental requirements.	Akerboom et al., 2012; Redolfi et al., 2021
	GCaMP6f/m/s	510	Able to detect single APs. 6f has shortest half-decay time. Variants can be chosen according to their properties to fit experimental requirements. Adequate for imaging experiments over multiple weeks. Adequate fluorescence at basal cell [Ca^2+^].	Chen et al., 2013; Rose et al., 2014; Redolfi et al., 2021
	jGCaMP7	515	Enhanced signal-to-noise ratio allows detection of individual spikes over wide fields of view (jGCaMP7s,f). Used for imaging of small processes thanks to the enhanced brightness (jGCaMP7b).	Dana et al., 2019; Redolfi et al., 2021
	jGCaMP8	∼510	Enhanced signal intensity, similar kinetics to GCaMP6. Well suited for bulk population recordings.	Ohkura et al., 2012b; Redolfi et al., 2021
RFP	RCaMP1a	630	Brighter than RGECO, but lower calcium binding affinity and dynamic range. Not activated by blue light, making it suitable for use with optogenetics.	Akerboom et al., 2013; Dana et al., 2016
	RCaMP1.07	584	Higher fluorescence than RGECO. Well suited for use with optogenetics.	Ohkura et al., 2012a
	RCaMP2	583	Strong single AP responses, very fast kinetics, suited for dual color calcium imaging with green fluorescent indicators.	Inoue et al., 2015
Multiple color variants	XCaMP	blue, green, yellow, red	Improved signal linearity. Adequate fluorescence at basal cell [Ca^2+^] (XCaMP-G). Enables measurement of fast-spike trains in individual neurons.	Inoue et al., 2019; Redolfi et al., 2021

**TABLE 3:** Non-GCaMP-based genetically encoded calcium indicators, including GECOs and camgaroos.

Class	Indicator	Emission wavelength (nm)	Description	References
GECO	G-GECO1-1.2	∼512	Better than GCaMP3 for monitoring spontaneous Ca2 + oscillations. Higher fluorescence than GCaMP3. pH-sensitive.	Zhao et al., 2011
	B-GECO1	446	Suited for multichromatic imaging. pH-insensitive compared to G-GECOs. Interference with mitochondrial autofluorescence.	Zhao et al., 2011
	R-GECO1	∼600	Greater dynamic range and higher calcium binding affinity than RCaMP. Susceptible to artifact-induced excitation with blue and green light.	Zhao et al., 2011; Akerboom et al., 2013
	NIR-GECO2/2G	685/40	Near-infrared calcium indicators susceptible to infrared light-induced activation. Lower brightness and slower kinetics than standard green and red GECIs.	Qian et al., 2020
	GEX-GECO1	∼510	Fast kinetics, large dynamic range.	Zhao et al., 2011
	GEM-GECO1	511/455	Good signal range, mitochondrial autofluorescence does not interfere with its activity.	Zhao et al., 2011
	jRGECO1a	630	Faster than RCaMP.	Dana et al., 2016
Camgaroos	Camgaroo-1	513	Unable to detect single APs. Exhibits slower kinetics than latest GCaMP versions.	Baird et al., 1999; Barth, 2007
	Camgaroo-2	535	Unable to detect single APs. Exhibits slower kinetics than latest GCaMP versions.	Griesbeck et al., 2001; Barth, 2007

In parallel with the continued development of diverse fluorescent indicator proteins, imaging technologies have also advanced. Classical GECI recordings were often made in superficial brain areas due to the relative ease of access for optical observation. The development of miniaturized fiber optic-based photometry has facilitated studying deep-brain structures in awake, behaving animals in real-time (Helmchen et al., 2001; Grewe et al., 2010; Lütcke et al., 2010). However, fiber photometry measures bulk calcium dynamics, lacking cellular resolution. Now, experiments using implantable gradient refractive index (GRIN) lenses combined with mini-microscopes or 2-photon interfaces permit real-time dynamic recordings from individual cells deep within the brain (Helmchen et al., 2001; Barretto et al., 2009; Ghosh et al., 2011; Zong et al., 2017; Zhang et al., 2019). GECIs represent a powerful tool for monitoring the activity of neural circuits in a cell type-specific manner. This highly optimized and titratable system has revolutionized the field of systems neuroscience, allowing for bulk activity recordings and spike extractions from big data sets such as the Machine Intelligence from Cortical Networks (MICrONS) data set, which contains large scale tracing and functional connectivity data obtained through electron microscopy (Zhou et al., 2020). The combination of highly optimized calcium indicators, genetic targeting technology, sophisticated imaging techniques, and big data analysis through machine learning approaches have opened the door for an in-depth, mechanistic understanding of circuit dynamics with unprecedented precision.

### Genetically-Encoded Voltage Indicators

While GECIs remains an invaluable tool to study cell-type-specific circuit dynamics, due to their dependence on calcium activity they represent an indirect measure of cell activity. The development of genetically encoded voltage indicators (GEVIs) directly measures neuronal activity, as indicated by neuronal membrane potential, in a more temporally precise manner than calcium indicators (Table 4). Although the advent of GEVIs predates GECIs until recently, circuit monitoring via GEVIs has suffered from limitations in imaging technologies and available voltage indicators. Advancements in image processing and GEVI development have paved the way for the visualization of voltage dynamics in genetically defined circuits (Chamberland et al., 2017). The first GEVIs were designed as fusion proteins between GFP and voltage-sensing domains (VSDs) like those found in voltage-gated ion channels (Siegel and Isacoff, 1997; Sakai et al., 2001; Dimitrov et al., 2007). Within the class of VSD-based GEVIs, two main families of indicators have been categorized by the number of fluorescent proteins fused to the VSD: FRET GEVIs (also known as ratiometric GEVIs) and single fluorescent protein (monochromatic) GEVIs.

**TABLE 4:** Commonly used GEVIs.

Class	Indicator	Fluorescence reporter (Emission wavelength nm)	Description	References
Ion Channel-FPs	Shaker Kv-based FlaSH	FLaSH-GFP (505)	Slow, cannot resolve APs. Potential co-assembly with native channel subunits. Low fluorescence change.	Jin et al., 2011; Kostyuk et al., 2019
	SPARC	GFP (505)	Faster than FLaSH-based sensors, reports pulses as low as 2ms. Does not inactivate during extended depolarizations. Low fluorescence change.	Ataka and Pieribone, 2002; Kostyuk et al., 2019
	FlaSH with split FP	Depends on FP	Low fluorescence change; slow kinetics.	Jin et al., 2011
FRET	VSFP1/2s, CR	CFP (477) YFP (529) citrine (529)	Reliable membrane targeting. Limited by slower response kinetics compared to VSFP butterflies.	Sakai et al., 2001; Dimitrov et al., 2007; Lundby et al., 2008; Mutoh et al., 2009; Lam et al., 2012; Mishina et al., 2014
	Chimeric VSFP butterflies	Citrine (542) mKate2 (594)	Faster than previous VSFPs. Reports membrane voltage oscillations up to 200 Hz.	Mishina et al., 2014
	Nabi	Nabi1: mKO (559), UKG (499); Nabi2: Clover (515), mRuby2 (600)	Better SNR, faster than VSFP butterflies and VSFP-CR.	Sung et al., 2015
Mono-chromatic FPs	VSFP3s	Citrine (529) mOrange (562) TagRFP (584) mKate2 (633)	VSFP3 faster than VSFP2.3. Red-shifted VSFP3 available.	Lundby et al., 2008; Perron et al., 2009
	Arclight	GFP (507)	Robustly reports AP and subthreshold events and reveals electrical signals in neurite branches.	Cao et al., 2013; Borden et al., 2017
	Bongwoori	s.e.pHluorin A227D (509)	Faster kinetics than Arclight. Resolves 60 Hz APs.	Piao et al., 2015
	CpFP-based VSFPs	Depends on the FP	Weaker fluorescence than red shifted indicators. However, the red shifted indicators are still slower and weaker compared to other VSFPs.	Gautam et al., 2009
	FlicR	cpmApple (560)	Bright and fast-read voltage indicator. Brighter than ArcLight Q239, similar kinetics to ArcLight Q239.	Abdelfattah et al., 2016
	ASAP family	CpGFP (509)	ASAP1 has on-off kinetics of ∼2ms, detects APs and subthreshold changes. Tracks AP trains up to 200 Hz. ASAP2 more sensitive, suited for 2p imaging.	St-Pierre et al., 2014; Chamberland et al., 2017
Rhodopsin fluorescent probes	Arch	Arch (687nm), Arch (D95N; 687nm)	Fast and sensitive voltage sensor but it is very dim.	Kralj et al., 2011
	QuasArs	QuasAr1 (H95Q) 715 nm	Both QuasAr1 and QuasAr2 have enhanced fluorescence compared to Arch (D95N).	Hochbaum et al., 2014
	Archons	Archon 1 retinal cofactor (637 nm)	Archon1 follows small, high-speed voltage changes in cultured neurons; brighter than QuasArs	Piatkevich et al., 2018
Opsin/FRET	Ace-mNeon	mNeonGreen (517 nm)	Responds five to six times as fast as MacQ-mCitrine and ASAP1 indicator; more photostable.	Gong et al., 2015
	VARNAM	MRuby3 N81S (565 nm)	Resolves APs with sensitivity and kinetics comparable to Ace-mNeon.	Kannan et al., 2018
	MacQ-m Citirine	mCitirine (529nm)	Faster kinetics than Arclight. Not brighter than Arclight but with a comparable or better SNR.	Gong et al., 2014
	QuasAr2-mOrange2	Retinal cofactor (715 nm)	Outperforms red-shifted ASAP1 and eFRET GEVIs in sensitivity, speed, SNR and photostability, but requires intense laser illumination.	Zou et al., 2014
Fret-Dye	Voltron	JF525 (532 nm)	Brighter than Ace2N-mNeon and ASAP2f in cell culture. Improved photostability compared to Ace2N-mNeon. In mice, allows single-trial recording of spikes and subthreshold voltage signals from dozens of neurons simultaneously for 15 min.	Abdelfattah et al., 2019
Other	hVOS	EGFP-F + DPA (505 nm)	Improved sensitivity and temporal resolution compared to previous GFP based probes.	Chanda et al., 2005
	VoltageSpy	(546 nm)	Robust, single-trial optical detection of APs at soma. Reports APs in axons and dendrites.	Grenier et al., 2019

*AP, action potential; SNR, signal-to-noise ratio. Knöpfel and Song (2019).*

Ratiometric GEVIs use voltage-dependent Förster resonance energy transfer (FRET) to modify the conformation of the VSD, in turn changing the conformation of two fluorescent proteins such that the readout is an anticorrelated switch from fluorescence in one color to another. FRET GEVIs are particularly useful for circuit monitoring applications *in vivo*, where the anticorrelated color-switching can be parsed out from hemodynamic correlated changes in fluorescents within the same color (Akemann et al., 2012, 2010). Monochromatic GEVIs, on the other hand, contain a single fluorescent protein that emits a single wavelength of light, best suited for cell culture or slice preparation experiments. The advantage of this kind of GEVI is that the optical imaging setup required for measuring its activity is less complex than that of the ratiometric GEVI. However, given that there is only a single wavelength emitted, performing *in vivo* imaging requires more complex corrective methods to account for hemodynamic changes and/or a multiplexed approach (Borden et al., 2017).

One of the major limitations of VSD-based GEVIs is the weak signal-to-noise ratio. Thus, a second major class of GEVIs was developed based on naturally occurring microbial opsins. These GEVIs take advantage of the native fluorescence of these opsins, but also amplify this signal by binding them to brighter fluorescent proteins, thus reducing the effect of photobleaching that is seen in other GEVIs (Isobe et al., 1983; Adam et al., 2019; Piatkevich et al., 2019). For example, FRET-opsins have been developed that couple rhodopsin to another fluorescent protein, such that the fast dynamics of the opsin can be coupled to the brightness of the additional fluorescent protein for better signal-to-noise ratios. Fluorescent proteins such as yellow mCitrine, the bright green mNeonGreen, or the orange mRuby3 and mScarlet, have been used to generate MacQ-mCitrine, Ace-mNeonGreen, VARNAM, and Ace-mScarlet, respectively (Gong et al., 2014, 2015; Gong, 2015; Kannan et al., 2018; Beck and Gong, 2019; Mollinedo-Gajate et al., 2021). These opsin-based GEVIs have been leveraged in the study of circuit dynamics in cell culture, slice preparations, and *in vivo* experiments within cerebellar Purkinje cells (Gong, 2015).

Förster resonance energy transfer-opsins represent an advancement in the design of GEVIs to overcome signal-to-noise limitations. However, due to a greater molecular size, they are susceptible to forming intracellular aggregates that impede their localization to the cell plasma membrane (Hochbaum et al., 2014; Kannan et al., 2019). To circumvent this limitation, a new class of chemigenetic hybrid GEVIs has been developed that consists of two components – the first which targets the indicator to a cell type of interest, while the fluorescent protein is separately delivered to the target tissue, often intravenously (Sundukova et al., 2019). Within the class of chemigenetic GEVIs, three main families of indicators exist: Opsin-Dye FRET chemigenetic indicators, fluorescent protein-dye FRET hybrid indicators, and indicators that use photo-induced electron transfer. The two most promising opsin-dye FRET chemigenetic indicators are Voltron and Positron – both of which use a microbial opsin fused to a self-labeling protein tag that allows for the binding of a fluorescent protein (Grimm et al., 2015; Abdelfattah et al., 2019, Abdelfattah et al., 2020). Fluorescent protein-dye FRET indicators use an exogenous chromophore coupled to a fluorescent protein, where the chromophore is used to quench fluorescence based on cell membrane voltage (Chanda et al., 2005; Wang et al., 2010). Lastly, photo-induced electron transfer GEVIs work using a voltage-sensitive fluorescent protein-tagged to the SpyTag protein which covalently binds to the SpyCatcher membrane protein in the cell (Grenier et al., 2019). Voltage sensing is achieved by photo-induced electron transfer through a synthetic wire in the membrane that reversibly quenches the activity of the fluorescent protein (Mollinedo-Gajate et al., 2021). The ability to target GEVI expression with cell-type specificity and improved membrane localization allows for greater reliability and precision in the quest to measure circuit dynamics.

Although fluorescent protein-based GEVIs represent a powerful way to measure neural activity, these indicators are limited in their photostability and potential for toxicity at high expression levels. A class of bioluminescent GEVIs has recently been developed that overcomes these limitations (Inagaki et al., 2019, 2017). Bioluminescent GEVIs provide cells with a substrate that can be converted into a fluorescent reporter based on changes in membrane potential. Traditional bioluminescent GEVIs combine a VSD, luciferase, and a fluorescent protein in a similar configuration to FRET-based GEVIs, whereby voltage changes produce a FRET-based anticorrelated signal between the luciferase and fluorescent reporter. While the anticorrelated design allows for better signal-to-noise ratios, it is limited by the requirement of the exogenous substrate, luciferin. To overcome this limitation, recent advancements have been made such that the bioluminescent GEVI self-generates an energy-rich substrate for light-emitting activity (Srinivasan et al., 2019; Mollinedo-Gajate et al., 2021).

While voltage indicator technology has advanced greatly in recent years, continued improvements to their photostability, signal intensity, and power of imaging platforms are required in order to more readily compete with calcium indicators (Yang and St-Pierre, 2016). However, new and improved GEVIs are continually being engineered to address these concerns, providing direct monitoring of electrical activity in living cells. Despite the high signal intensity provided by GECIs, this tool can only provide binary information on whether a neuron is active, lacking the temporal dynamics required to accurately determine neuronal firing patterns. Additionally, GECIs rely on indirect measures of neuronal activity via calcium. In contrast, GEVIs provide the necessary kinetics to accurately portray complex firing patterns, and even signal propagation throughout an individual neuron, but lack the appropriate signal intensity to generate high-quality data. A paradigm shift in the use of GECIs versus GEVIs may come in the future, but this requires continued advancements in both imaging techniques and protein engineering to improve GEVIs.

### Neurotransmitter and Neuropeptide Sensors

Electrophysiology, GECIs, and GEVIs reveal detailed neuronal activity, and with the power of viral and transgenic targeting technologies, interrogate circuit dynamics with cell-type specificity. However, these techniques are limited in their ability to measure cell-cell communication dynamics at the synapse. To study the real-time communications between active neurons, novel protein-based sensors that bind to and report the release of neurotransmitters have emerged, providing an unprecedented view of the dynamics of communication between neurons (Table 5).

**TABLE 5:** Properties and applications of genetically encoded neurotransmitter sensors.

Sensor	Excitation/Emission wavelengths (nm)	Detection range	ON kinetics (ms)	Applications *in vivo*	References
**Glutamate sensors**					
iGluSnFR	490/510	1 μM–10 mM	15	*C. elegans*, zebrafish, mouse	Marvin et al., 2013; Helassa et al., 2018; Helassa et al., 2018
SF-iGluSnFR-A184S	490/510	1 μM–10 mM	85	Mouse, ferret	Marvin et al., 2018
iGlu_u_	490/510	10 μM–10 mM	0.7	N/A	(Helassa et al., 2018
SF-Venus-iGluSnFR	515/528	1 μM–10 mM	N/A	Mouse	Marvin et al., 2018
R-iGluSnFR1	562/588	1 μM–10 mM	N/A	N/A	Wu et al., 2018
**GABA sensor**					
iGABASnFR	485/510	1 μM–10 mM	∼25	Zebrafish, mouse	Marvin et al., 2019
**Cholinergic sensors**					
iAchSnFR	485/510	0.1–100 μM	∼25	Zebrafish, mouse, *C. elegans*, *Drosophila*	Borden et al., 2020
iNicSnFR	485/535	1 μM–10 mM	∼1000	Zebrafish	Shivange et al., 2019
GACh3.0	492/510	0.1–100 μM	312	Mouse, *Drosophila*	Jing et al., 2020
**Serotonin sensors**					
iSeroSnFR	490/512	330 pM–5 mM	0.5	Mouse	Unger et al., 2020
sLight1.3	490/516	1 nM–10 μM	N/A	Mouse	Patriarchi et al., 2018
GRAB_5–HT_	490/510	1 nM–1 μM	200	Mouse	Wan et al., 2021
**Dopamine sensors**					
dLight1.1	490/516	10 nM–10 μM	10	Mouse	Patriarchi et al., 2018
dLight1.2	490/516	10 nM–10 uM	9.5	Mouse	Patriarchi et al., 2018
dLight1.3b	490/516	100 nM–100 μM	N/A	Mouse, rat	Patriarchi et al., 2018
dLight1.4	490/516	1 nM–1 μM	N/A	Mouse, rat	Patriarchi et al., 2018
GRAB_DA1m_	490/510	10 nM–1 μM	80	Mouse, *Drosophila*, zebrafish	Sun et al., 2018
GRAB_DA1h_	490/510	1 nM–10 μM	110	Mouse, *Drosophila*, zebrafish	Sun et al., 2018
R-dLight1	562/588	0.01–100 μM	14	Mouse, rat	Patriarchi et al., 2020
**Norepinephrine sensors**					
nLight1.3	490/516	0.1–100 μM	N/A	Mouse	Oe et al., 2020
GRAB_NE1m_	490/510	0.1–100 μM	72	Mouse, zebrafish	Feng et al., 2019
**Orexin sensors**					
OxLight1	470/560	0.1–10 μM	500–700	Mouse	Duffet et al., 2022
**Oxytocin sensors**					
MTRIA_OT_	490/510	0.01–1 μM	12,000	Mouse	Ino et al., 2021
GRAB_OT1.0_	490/510	0.001–1 μM	500	Mouse	Qian et al., 2022

*Adapted from Sabatini and Tian (2020).*

Neurotransmitter sensors come in two main classes – periplasmic binding protein (PBP)-based sensors and G-protein coupled receptor (GPCR)-based sensors. PBP-based sensors use microbial PBPs fused to fluorescent proteins to report the presence of neurotransmitters in the synaptic cleft. One of the first PBP-based sensors developed was iGluSnFR, which has been used to detect glutamate *in vitro* and *in vivo* in mice, worms, and zebrafish (Marvin et al., 2013). Since then, advanced versions of iGluSnFR with different color and kinetic properties have been developed. Other sensors in the PBP class of neurotransmitter sensors include iGABASnFR, iAChSnFR, and iSeroSnFR, as well as small molecule sensors like iATPSnFR, and iNicSnFR (Lobas et al., 2019; Marvin et al., 2019; Shivange et al., 2019; Borden et al., 2020; Unger et al., 2020). One caveat to using PBP-based sensors is that since they are microbially based, they may interfere with endogenous cellular processes and machinery.

GPCR-based sensors overcome this limitation, as they mimic the properties of endogenous receptors and are quite stable. GPCR sensors bind to neurotransmitters present in the synapse and cause a conformational change that results in a fluorescent signal. Some of the neurotransmitter sensors in this class include the dLight1 and GRAB_DA_ families of dopaminergic sensors as well as the GRAB_NE_, GRAB_5–HT_, and GRAB_ACh_ sensors for norepinephrine, serotonin, and acetylcholine, respectively (Jing et al., 2018; Patriarchi et al., 2018; Sun et al., 2018; Feng et al., 2019; Wan et al., 2021). Furthermore, the advent of neuropeptide GPCR-based sensors such as OxLight1, MTRIA_OT_, and GRAB_OT1_._0_ for orexin and oxytocin, respectively, permits the visualization of volume transmission from dendrites as well as axon terminals (Ino et al., 2021; Duffet et al., 2022; Qian et al., 2022). However, due to competitive interference from endogenous receptors, the readout of neurotransmitter activity using GPCR sensors will necessarily be based on the relative change in fluorescence rather than the absolute number of neurotransmitter molecules present at the synapse. Additionally, GPCR-based sensors cannot be used for pharmacological studies as they will interfere with the drug-receptor interactions. Thus, depending on the application, either PBP or GPCR sensors may be preferred.

The development of neurotransmitter sensors heralds a new era of neuroscientific discovery, where cell-cell neuronal communication is not only capable of being monitored at the cellular level, but at the synapse itself. In fact, biosensors are even being developed to monitor the activity patterns of second messengers downstream of synaptic transmission (Tewson et al., 2016; Massengill et al., 2021). For example, recent studies using a cAMP biosensor revealed that dopamine-triggered cAMP release in the basal amygdala facilitates learning (Lutas et al., 2022). With the advent of these sensors, neurotransmitter and signaling cascade dynamics may be studied to understand how these molecules play a role in the development and function of circuit architecture within the brain. Additionally, when used in tandem with GECIs or GEVIs, one may monitor both neural activity and neural signaling dynamics at once.

## Manipulating Neural Circuits: Gain-And Loss-Of-Function Approaches Toward Interrogating Circuit Function

Mapping circuit connectivity and monitoring its activity patterns reveal critical knowledge regarding brain anatomy and function. However, monitoring a circuit even with cell-type-specific genetic tools only provides correlative data on circuit function. To test hypotheses, researchers often implement gain- or loss-of-function experimentation. About 200 years ago, through post-mortem observation of diseased patients, Dr. Broca identified a lesioned area in the left frontal cortex of an individual that contributed to speech production, as this patient had speech defects (Broca, 1861). While lesioning may still be used in neural systems research, the ability to draw correlations between brain structure and function has accelerated substantially in the last several decades due to the advent of genetically targeted gain- or loss-of-function strategies. Activating a circuit or node reveals its sufficiency to drive a particular phenotype. Reciprocally, inhibiting a circuit or node reveals whether it is necessary for a particular phenotype. Redundant and interdependent circuits add a layer of complexity to these experiments, as they may reveal a circuit to be only sufficient, only necessary, or both toward a particular phenotype. Recent advances in targeted genetic approaches have dramatically improved our ability to manipulate circuits with greater genetic, spatial, and temporal specificity.

### Targeted Ablation

Although electrolytic or chemical lesioning methods remain powerful techniques to interrogate structure and function relationships in the brain (Foster et al., 2003; Lavond and Steinmetz, 2003), these approaches are non-specific, ablating all the cells in a given area. Alternatively, genetically-targeted ablation may be used for cell type-specific lesioning. One such system was adopted from the bacteria *Corynebacterium diphtheriae*. Its earliest rendition involved the use of transgenic lines that placed a diphtheria toxin (DT) gene under a promoter of interest (Breitman et al., 1987; Saito et al., 2001). Cells that used this promoter would express DT, driving their own death. The principal limitation of this technology was that the timing of cellular ablation could not be controlled. Since ablating cells early in development may result in compensatory plasticity mechanisms, it became desirable to temporally control ablation. In 2001, these limitations were abrogated by controlling cellular expression of the DT receptor (DTR), which is not endogenously expressed in the mouse. In this way, the experimenter could selectively express DTR in specific cell types, and provide exogenous DT at any point in development to selectively ablate cells expressing DTR (Saito et al., 2001). This technology led to the discovery that AgRP/NPY neurons are required for proper feeding behaviors in adult mice, but are not required in neonate mice, perhaps due to compensatory mechanisms (Luquet et al., 2005). One important consideration with cellular ablation is that it is permanent. Other experiments may require reversible manipulation, such as the techniques discussed below.

### Pharmacology

Neuronal activity can be manipulated by specific chemicals. Commonly, this is due to agonistic or antagonistic effects on neural ion channels (Narahashi et al., 1964; Collingridge et al., 1983; Sweeney et al., 1995; Verderio et al., 1999; Corcoran and Quirk, 2007). By infusing pharmacological substances in particular brain regions, it is readily possible to investigate the effect of activating or inhibiting targeted areas. One common means of drug delivery is through a stereotaxically implanted hollow cannula (Sike et al., 2017). This can be combined with other technologies such as microdialysis or *in vivo* imaging to dissect the effects of drug treatments on neurotransmitter release, neural activity, and gene expression. Commonly used pharmacologically active chemicals used to manipulate neuronal activity are summarized in Table 6. Importantly, pharmacological manipulations are specific to cells that express a particular receptor but are not selective in the cell types they may target. For reversible and cell type-specific manipulation experiments, opto- or chemogenetics have proven invaluable.

**TABLE 6:** Commonly used pharmacological agents in neural circuit studies.

Compound	Description	References
Tetrodotoxin (TTX)	Puffer fish-derived chemical that selectively blocks voltage gated sodium channels most associated with the action potential. Most useful in electrophysiologic confirmation of a monosynaptic connection between neurons. *In vivo* it can also be used to inhibit brain regions through cannulation.	Narahashi et al., 1964
APV	Blocks the NMDA glutamate receptor. It has been critical in understanding both long term depression and potentiation.	Collingridge et al., 1983
CNQX	A stable and selective blocker of AMPA receptors, drawn from a broad class of engineered compounds known as quinoxalinediones.	Corcoran and Quirk, 2007
Tetanus toxin	A bacterially derived protein that can inhibit SNARE mediated exocytosis, thereby inhibiting neurotransmission. In 1995, this technology was made to be expressed directly by neurons, paving the way for current cell-type specific vesicular release silencing.	Sweeney et al., 1995; Verderio et al., 1999

### Optogenetics

In the late 1970s, Francis Crick remarked that the real challenge of neuroscience would be to precisely control the activity of a desired set of neurons. Nearly three decades later, George Nagel, Peter Hegemann, and colleagues discovered Channelrhodopsin-2 (ChR2), a light-gated cation channel that demonstrated inward cationic current with millisecond time-scale resolution (Nagel et al., 2003). Shortly after this discovery, Boyden, Deisseroth, and colleagues showed that ChR2 could be leveraged to manipulate the activity of neurons using light in a technique known as optogenetics (Boyden et al., 2005). Currently, it remains one of the most temporally and spatially specific methodologies to manipulate neural activity, both *ex vivo* and *in vivo*, in which mice are implanted with fiber optics for light delivery (Yizhar et al., 2011). Optogenetics has led to many systems-level discoveries, such as the solidification of arcuate hypothalamic feeding circuitry. In this study, conditional expression of ChR2 and subsequent activation of AgRP cells in the arcuate nucleus of the hypothalamus led to robust acute food consumption, while optogenetic activation of POMC neurons led to decreased food consumption (Aponte et al., 2011).

**FIGURE 6:**
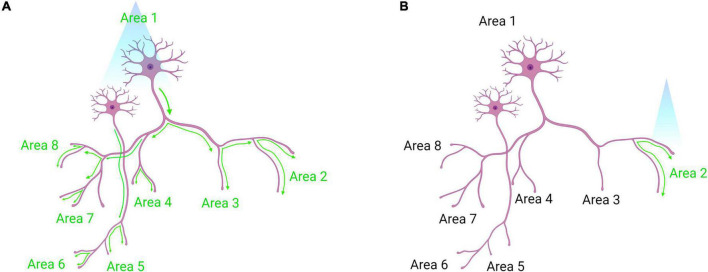
Somatic optogenetic stimulation versus terminal field optogenetic stimulation. **(A)** Optogenetic stimulation of a population of neurons at their soma will lead to downstream activation of all the projection targets of that population (as indicated by green activation arrows). **(B)** Terminal field stimulation using optogenetics. Here, an investigator may be interested in the function of Area 1 neuron projections to Area 2 specifically. By placing the laser directly above Area 2, the investigator will activate only those projection terminals to dissect the neural circuit function more precisely. Created with Biorender.com.

At the core of optogenetics is the use of opsins. Upon photon absorption, opsins undergo a conformational change that allows for ion transport across the plasma membrane, either depolarizing or hyperpolarizing neurons (Foster and Bellingham, 2002). As described in Table 7, a plethora of opsins has been engineered that allow great flexibility in their experimental use. These opsins have attributes that differ in terms of effects on membrane potential, kinetics, and absorption spectra (Yizhar et al., 2011; Deisseroth, 2015). Furthermore, more than one type of opsin can be expressed by cells. For example, an excitatory opsin activated by blue light (e.g., ChR2) can be co-expressed with an inhibitory opsin activated by yellow light (e.g., halorhodopsin; Han and Boyden, 2007). This allows for either gain- or loss-of-function experimentation within the same animal. Another consideration in the design of an optogenetic experiment is the location of the light source, either at the soma or axon terminals of the opsin-expressing neural population. If placed at the soma, those cells and all of their projection targets will be affected (Figure 6A). If placed at the axon terminals, only that projection site will be affected (Yizhar et al., 2011; Figure 6B). The latter approach allows for greater dissection of the exact circuitry involved in a particular phenotype (Steinberg et al., 2014). Interestingly, stimulating somata versus terminal fields can result in disparate phenotypes. For example, one study used opsins to stimulate or inhibit basolateral amygdala (BLA) projections to the central amygdala, which altered anxiety-related behaviors, while stimulating BLA cell bodies had no effect. This was perhaps due to downstream projections operating in opposition to each other (Tye et al., 2011). Thus, terminal field stimulation is very useful for dissecting circuit function, as cells may have diverse collateralizations.

**TABLE 7:** Opsins are commonly used in optogenetics with their salient properties.

Effect	Variant	Description of opsin	Peak activation wavelength (nm)	References
Excitatory (de-polarizing)	ChR2	Most widely used cation conducing opsin, non-specific cation channel from *Chlamydomonas reinhardtii*	470	Boyden et al., 2005
	ChR/T159C and ChR2/H134	Mutations in ChR2 that induce relatively larger photocurrents.	450, 470	Lin et al., 2009; Berndt et al., 2011
	ReaChR	Red-shifted variant of ChR2.	590	Lin et al., 2013
	ChETA (E123T)	ChR2 mutations that induce more rapid kinetics, at the cost of smaller photocurrents.	490	Lin, 2011
	SFO/SSFO	Step-opsins with delayed closing of ion channel for sustained action (current will continue upon cessation of light); quickly closes with red-light pulse.	470 (closed with 590)	Berndt et al., 2009
	ChrimsonR	Red-shifted variant of ChR2.	590	Klapoetke et al., 2014
	C1C2GA	Blue-shifted microbial opsin-based variant.	455	Kato et al., 2015
Inhibitory (hyper-polarizing)	Halorhodopsin (eNph3.0)	Light-gated chloride channel found in halobacteria. Red-shifted, improved trafficking to membrane.	589	Gradinaru et al., 2010
	Archaerhodopsins (eArch3.0, eArchT 3.0)	Light gated proton pump from Halobrum. ArchT 3.0 has enhanced trafficking to membrane and light sensitivity.	566	Mattis et al., 2011
	Jaws	Red shifted cruxhalorhodopsin from *Haloarcula*; acts as a chloride pump.	632	Chuong et al., 2014
	SwiChR(CA)	A mutated chimera of ChR that acts as a chloride-conducting anion channel; delayed off-kinetics rapidly corrected by brief red light pulse	475	Berndt et al., 2014
	iChlocC	Mutant of ChR that permits chloride conduction with high selectivity and sensitivity	476	Wietek et al., 2015
	GtACR1, 2, and ZipACR1	Light-gated chloride channels from *Guillardia theta*	ACR1: 515 ACR2: 470 Zip: 520	Govorunova et al., 2015, 2017
	Aurora	Engineered anion-conducting channelrhodopsin; Red-shifted, with step-function capabilities	517	Wietek et al., 2017
Dual color actuators	BiPOLES	Dual color controller of neuronal activity that allows for excitation or inhibition based on photostimulation wavelength.	Inhibition: 490 Excitation: 635	Vierock et al., 2021

However, it must be considered that the nature of optogenetics frequently necessitates a level of invasiveness that may be unacceptable. For example, very deep brain regions with functions essential to survival may not be compatible with external light delivery (Chen et al., 2021). Further, the tethering required by most light-delivery systems may compromise the validity of certain behavioral experiments (Won et al., 2020). Finally, light delivery may lead to tissue toxicity, for example, due to heating or ischemia (Chen et al., 2017a). Therefore, at times less invasive means may be needed to manipulate neural circuits transiently, which is possible through chemogenetics.

### Chemogenetics

Chemogenetics refers to a set of techniques whereby genetically engineered receptors expressed on cell surfaces bind to exogenously delivered ligands. Importantly, these ligands are not endogenously produced. Early examples of chemogenetic technologies came in 1998 with Receptors Activated Solely by a Synthetic Ligand (RASSLs; Conklin et al., 2008). Currently, the most commonly used receptors for manipulations are coupled to G proteins known as DREADDs: Designer Receptors Exclusively Activated by Designer Drugs. DREADDs are activated by the ligand Clozapine-N-Oxide (CNO; Whissell et al., 2016; Table 8). Depending on the receptor’s associated downstream signaling cascade, the ligand may be excitatory (Gq, Gs) and increase cell firing, or inhibitory (Gi) and decrease cell firing. While CNO-activated DREADDs are one of the most widely used tools for chemogenetic neural manipulation, this technology has some major limitations. It has been shown that CNO does not cross the blood-brain barrier, but rather its parent metabolite, clozapine (a known antipsychotic drug), exerts effects in the brain (Gomez et al., 2017). Additionally, clozapine has been shown to bind to endogenous receptors throughout the body at high doses and with known effects on animal behavior (Desloovere et al., 2019). However, these findings remain controversial, as other studies report opposing findings in mice (Guettier et al., 2009; Jendryka et al., 2019). To overcome such limitations, studies have shown that at low doses of clozapine, these off-target effects are reduced (Manvich et al., 2018; Matsui et al., 2018). Additionally, novel ligands like olanzapine, JHU37160/152, and deschloroclozapine are being used for DREADD-based manipulations as they have a minimal conversion to other compounds and less off-target effects (Bonaventura et al., 2019; Weston et al., 2019; Nagai et al., 2020).

**TABLE 8:** Common chemogenetic receptors and ligands with their associated properties and effects on neuronal activity.

Receptor name	Mechanism of action	Effect on neuronal firing	Ligand	References
hM3Dq	Phospholipase C Dependent (activator)	Increased	CNO/clozpine/JHU37152/ JHU37160/deschloroclozapine (DCZ)	Alexander et al., 2009; Gomez et al., 2017; Bonaventura et al., 2019; Nagai et al., 2020
hM1Dq	Phospholipase C Dependent (activator)	Increased	CNO	Alexander et al., 2009
hM5Dq	Phospholipase C Dependent (activator)	Increased	CNO	Armbruster et al., 2007
hM4Di	Adenylate Cyclyase Dependent (inhibitor)	Decreased	CNO/clozpine/JHU37152/ JHU37160/deschloroclozapine (DCZ)	Zhu et al., 2014; Gomez et al., 2017; Matsui et al., 2018; Bonaventura et al., 2019; Nagai et al., 2020
hM2Di	Adenylate Cyclyase Dependent (inhibitor)	Decreased	CNO	Armbruster et al., 2007
Rq(R165L)	B-arrestin dependent	unknown	CNO	Nakajima and Wess, 2012
KORD	Adenylate Cyclyase Dependent (inhibitor)	Decreased	Salvinorin B	Vardy et al., 2015
PSAM-5HT3	Ligand gated cation channel	Increased	varinicline	Magnus et al., 2019
PSAM-GlyR	Ligand gated anion channel	Decreased	varinicline	Magnus et al., 2019

Some experiments require one to manipulate several neural populations at once or to bidirectionally control the activity of one neural population. Through a recently developed technology labeled DREADD KORD(R), chemogenetics is afforded the possibility of multiplexing. DREADD KORD(R)s are sensitive to a distinct exogenously delivered ligand known as Salvinorin B and completely insensitive to CNO (Vardy et al., 2015). The non-overlapping pharmacologic activation of DREADD KORD(R) and DREADDs provides superior bidirectional manipulation relative to optogenetics, since opsins may absorb photons at overlapping ranges, potentially confounding results. However, the temporal resolution of DREADD action is less precise than optogenetics and operates on the timescale of minutes to hours (Whissell et al., 2016). This is because it takes time for the synthetic ligand (e.g., CNO) to distribute to areas that express the DREADD, and also because GCPRs have slower kinetics than ion channels. This can be beneficial when requiring more chronic forms of manipulation, but less useful for applications where faster or more temporally-precise manipulations are desired.

While chemogenetics is limited by less precise temporal and spatial resolution, newer technologies are emerging. For example, magnetically-sensitive lipid nanoparticles carrying CNO ensure the steady release of CNO to targeted circuits (Rao et al., 2019). Additionally, it is possible to employ both opsins and DREADDs for bidirectional control of neural activity (Han et al., 2017; Siemian et al., 2021). Alternatively, the development of Pharmacologically Selective Activator Modules (PSAMs) which are activated by Pharmacologically Selective Effector Molecules (PSEMs) increases temporal resolution (Shapiro et al., 2012). Unlike DREADDs which alter neuronal activity through the modulation of GPCR-biased signaling cascades, PSAMs affect neuronal activity through modulation of ligand-gated ion channels. Most recent iterations of the technology use the agonist varenicline, an FDA-approved drug. Although varenicline binds to nicotinic receptors (ligand-gated ion channels), scientists have generated a PSAM that binds to varenicline at a low enough concentration that it will not bind to endogenously produced receptors (Magnus et al., 2019).

Both optogenetic and chemogenetic manipulations allow for controlled activation or inhibition of circuitry in a genetically-defined manner. While useful, these technologies are limited by the need to consistently deliver an actuator (e.g., light through fiber optics for optogenetics or ligands for chemogenetics). Thus, using opto- or chemogenetics for studies in which a circuit would need to be activated/inhibited for more than several hours each day (for example, body weight or metabolism studies) is not optimal. For these studies, it is desirable to chronically activate neural activity, as with the overexpression of exogenous ion channels (Johns et al., 1999; Ren et al., 2001; Lin et al., 2010; Patel et al., 2019; Zhu et al., 2020).

### Ion Channel Manipulations: Kir and NaChBac

Neuronal excitability corresponds to a neuron’s likelihood of triggering an action potential. This likelihood is dependent on ion channel expression and functionality. Given the same stimuli, neurons with greater excitability will fire more frequently than neurons with lesser excitability (Hille, 2001). Scientists can bias excitability by manipulating the types and amounts of ion channels expressed on the neuronal plasma membrane so as to shift resting membrane potential (Johns et al., 1999; Lin et al., 2010; Zhu et al., 2020). Since these methods do not require drug administration or photostimulation, they are less invasive measures for studying neural function. However, these methods lack precise on/off control compared to optogenetics or chemogenetics.

One ion channel used to manipulate neural activity chronically is Kir2.1, an inward rectifying potassium channel that makes action potential generation more difficult (Johns et al., 1999). In 2017, the targeted expression of Kir2.1 helped to elucidate a circuit node responsible for integrating social input and modulating aggressive behavioral output (Watanabe et al., 2017). In 2020, the technology was used to understand the arcuate neuronal contribution to feeding behaviors (Zhu et al., 2020). To increase the temporal flexibility of this technology, methods have been devised to temporally control the expression of the channels using ligands or light, or both. For example, SPARK (Synthetic Photoisomerizable Azobene Regulation K + channels) has a genetically engineered K + channel that is sensitive to an exogenously delivered, light-sensitive ligand (Banghart et al., 2004). In the absence of the ligand, the channel remains open and hyperpolarizes the cell. In the presence of the ligand, and the absence of UV light, the K + channel closes. Finally, in the presence of both the ligand and UV light, the channel opens. This affords the flexibility of choosing when to hyperpolarize the cell or allow resting neuron conditions.

Other exogenous ion channels may be used to increase cell excitability and provide gain-of-function approaches. One such channel is the voltage-gated Sodium Bacterial Channel (NaChBac; Ren et al., 2001; Lin et al., 2010). Most importantly, NaChBac opens at more negative voltages relative to eukaryotic sodium channels. Consequently, subthreshold stimuli open these channels, thereby conferring both a greater depolarizing stimulus and an increased probability of action potential generation. Secondly, upon opening, they enable long-lasting depolarizing stimuli that can drive further action potentials. NaChBacs have been used to understand the roles of both corticotropins-releasing hormone (Zhu et al., 2020) and glutamate (Herman et al., 2016; Patel et al., 2019) in modulating feeding behaviors.

### Mapping and Monitoring Through Manipulation

Several experimental approaches combine diverse manipulation methods, such as optogenetics, with viral tracing or circuit monitoring techniques. An elegant intersection between circuit mapping, monitoring, and manipulation is the technique known as Channelrhodopsin Assisted Circuit Mapping (CRACM; Petreanu et al., 2007). This technology is a gold standard in the generation of reliable, functional circuit diagrams. Here, opsins and reporters are expressed on a pre-synaptic cell of interest. Then, by taking whole-cell recordings of a putative post-synaptic cell while stimulating the pre-synapse, one may identify synaptic currents on the post-synaptic cell. The temporal coincidence between light stimulation and post-synaptic currents, combined with the application of pharmacological agents that abolish this response, provides strong evidence for a functional synaptic connection between pre- and post-synaptic cells (Petreanu et al., 2009). While CRACM is useful, depending on the circumstance, it can have drawbacks. For example, depending on the expression profile of the opsin, it can be difficult to determine which side of the pre-or post-synapse is being excited by light when an electrophysiological event is being recorded in the post-synaptic cell. This can be mitigated by conditionally expressing the opsin in a neuronal subtype distinct from the post synaptic neuron. Alternatively, one can use an opsin whose expression is spatially restricted to the soma/proximal dendrites (Baker et al., 2016). One other limitation is the challenge of performing CRACM with peptidergic cell types. This is because neuropeptides often signal via volume transmission, which occurs over large distances and with potentially slower effects compared to fast neurotransmitters (like glutamate and GABA; Fuxe et al., 2013; Nederpelt et al., 2016).

**FIGURE 7:**
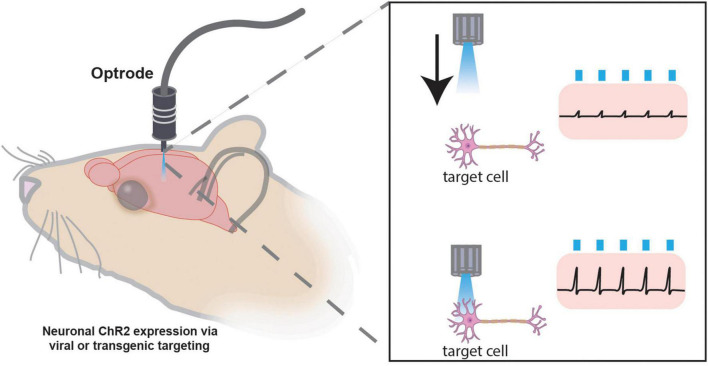
Optogenetic “sonar” method for targeting specific cellular populations for *in vivo* electrophysiology. Photostimulation “sonar” guides optrode to genetically targeted neurons expressing channelrhodopsin (ChR2) for extracellular electrophysiology recordings. Once the electrode is juxtaposed to a ChR2-expressing cell of interest, electrophysiological recordings may be performed. The optrode may have one or many channels for recording numerous cells simultaneously.

Without sophisticated techniques like Patch-Seq, electrophysiology lacks the ability to identify the exact cells being recorded. Combining electrophysiology with optogenetics allows *in vivo* targeting of genetically labeled cells for electrophysiological recordings through a “sonar”-like method (Lima et al., 2009; Hangya et al., 2015). The development of small implantable optrodes, or electrode(s) juxtaposed to a fiber optic for light delivery, locates conditional ChR2-expressing neurons via optogenetic stimulation *in vivo.* Once ChR2-expressing cells of interest are located (as indicated by reliable light-induced single units), extracellular recordings assess cellular electrical activity (Figure 7). The “opto-sonar” technique provides a dependable method to genetically identify and target circuits within a live, freely behaving animal. Indeed, with the integration of optogenetics and implantable MEAs, it is now commonplace to not only monitor the activity of genetically defined circuits but manipulate and assess their activity and temporal dynamics *in vivo* (Kim C. K. et al., 2017).

Another emergent multiplexed technology is known as holographic imaging. Holograms are light fields that form any simple and complex shape through wavefront interference. By combining imaging technologies with principles of holography, it has become possible to map, monitor, and manipulate populations of neurons in multiple regions of interest (in different focal planes) with an unprecedented combination of spatial and temporal resolution (Quirin et al., 2014; Yang et al., 2015). In a 2018 paper, Yang and colleagues simultaneously photostimulated 80 neurons within the mouse visual cortex while also monitoring the neighboring neuronal activity (Yang et al., 2018). This technology has since been leveraged to understand how neural ensembles within cortical circuitry can drive recall in a complex behavioral task (Carrillo-Reid et al., 2019). While certain technical limitations to the technology exist (e.g., limited field of view, lag in modulating projected holographic patterns, etc.), more profound challenges present themselves in how the output data is analyzed and interpreted (Carrillo-Reid et al., 2017; Yang and Yuste, 2018). To better use this emergent technology, creating methods to analyze these huge datasets must be a major focus moving forward.

## Discussion

The advent of genetic technologies has provided much insight into the complexities of how neural circuits are anatomically and functionally connected. However, dissecting circuits even at the level of molecularly defined groups of neurons may soon be too general. Thus, we expect the future circuit analyses will be done at the level of an individual cell, leveraging omics-based high-throughput approaches to map, monitor, and manipulate neural circuits. For example, while numerous cell-type-specific Cre and Flp drivers have been developed in recent years, continued advances in single-cell sequencing to generate novel drivers, and the use of intersectional and activity-driven genetic labeling techniques may afford the ability to isolate neural subpopulations with greater specificity. Additionally, multiplexed viral tracing techniques strategies that contribute toward maps with greater definition will provide rich anatomical datasets compared to traditional viral tracing or tract labeling methods. Such multiplexed approaches combined with tissue contemporary tissue clearing methods will facilitate the exquisite ability to map individual cells within complex circuits and in three dimensions. Collectively, these methods will rapidly advance the building of comprehensive circuit wiring diagrams with greater resolution.

While electrophysiology will remain a standard for evaluating synaptic connectivity and measuring the electrical properties of individual neurons, novel high-throughput electrophysiology approaches that enable simultaneous recording of hundreds of neurons at once, as with Neuropixels, greatly surpasses current electrophysiology limitations. Additionally, optical approaches for monitoring circuit dynamics will continue to evolve and expand. For example, genetically encoded calcium and voltage indicators, as well as neurotransmitter and neuropeptide sensors, will most likely steadily improve in signal-to-noise, photostability, and kinetics, thus expanding their utility greatly. When used in tandem with GRIN lenses or 2-photon imaging capabilities, genetically encoded imaging experiments can provide a clear picture of the signaling dynamics of individual neurons within dynamic networks. Indeed, the toolbox for circuit activity manipulations will continue to expand as well, with continued advances in optogenetics, chemogenetics, and genetically engineered ion channels with improved temporal, spatial, and cell type precision. With the help of *in silico* and directed evolution protein engineering methodologies, this will allow a cornucopia of different approaches toward mapping and manipulating neural circuits within living brain tissue.

In the future, multiplexing of a variety of approaches to map, monitor, and manipulate circuits simultaneously will provide more precise experimentation than current methods. However, the major limitations we currently face with these multiplexed, omics-based, and high-throughput methodologies is how these huge datasets will be processed and analyzed in order to make meaningful conclusions about the brain. This will require computation scientists, bioinformaticians, and neuroscientists to work together toward solving these unprecedented problems. Ultimately, better-informed circuit diagrams that link anatomy with the function will provide novel therapeutic targets for numerous neurological diseases, which may include obesity/eating disorders, psychiatric disorders, and neurodegenerative diseases.

## Author Contributions

JS, P-SC, JR, and SS: conceptualization, writing, editing, and figure and table generation. PH: writing, figure and table generation. JO-G: editing, figure and table generation. BA: conceptualization and editing. All authors contributed to the article and approved the submitted version.

## Conflict of Interest

The authors declare that the research was conducted in the absence of any commercial or financial relationships that could be construed as a potential conflict of interest.

## Publisher’s Note

All claims expressed in this article are solely those of the authors and do not necessarily represent those of their affiliated organizations, or those of the publisher, the editors and the reviewers. Any product that may be evaluated in this article, or claim that may be made by its manufacturer, is not guaranteed or endorsed by the publisher.
